# Association between age at onset of multimorbidity and incidence of dementia: 30 year follow-up in Whitehall II prospective cohort study

**DOI:** 10.1136/bmj-2021-068005

**Published:** 2022-02-02

**Authors:** Céline Ben Hassen, Aurore Fayosse, Benjamin Landré, Martina Raggi, Mikaela Bloomberg, Séverine Sabia, Archana Singh-Manoux

**Affiliations:** 1 Université de Paris, Inserm U1153, Epidemiology of Ageing and Neurodegenerative Diseases, Paris, France; 2 Department of Epidemiology and Public Health, University College London, London, UK

## Abstract

**Objective:**

To examine the association of midlife and late life multimorbidity, including severity of multimorbidity, with incident dementia.

**Design:**

Prospective cohort study.

**Setting:**

Civil service departments in London (Whitehall II study, study inception in 1985-88).

**Participants:**

10 095 participants, aged 35 to 55 at baseline.

**Main outcome measure:**

Incident dementia at follow-up between 1985 and 2019. Cause specific Cox proportional hazards regression was used to examine the association of multimorbidity overall and at age 55, 60, 65, and 70 with subsequent dementia, taking into account the competing risk of death.

**Results:**

The prevalence of multimorbidity (≥2 chronic diseases) was 6.6% (655/9937) at age 55 and 31.7% (2464/7783) at age 70; 639 cases of incident dementia occurred over a median follow-up of 31.7 years. After adjustment for sociodemographic factors and health behaviours, multimorbidity at age 55 was associated with subsequent risk of dementia (difference in incidence rate per 1000 person years 1.56, 95% confidence interval 0.62 to 2.77; hazard ratio 2.44, 95% confidence interval 1.82 to 3.26). The association weakened progressively with older age at onset of multimorbidity. At age 65, onset of multimorbidity before age 55 was associated with 3.86 (1.80 to 6.52) per 1000 person years higher incidence of dementia (hazard ratio 2.46, 1.80 to 2.26) and onset between 60 and 65 was associated with 1.85 (0.64 to 3.39) per 1000 person years higher incidence (1.51, 1.16 to 1.97). Severity of multimorbidity (≥3 chronic diseases) at age 55 was associated with a 5.22 (1.14 to 11.95) per 1000 person years higher incidence of dementia (hazard ratio 4.96, 2.54 to 9.67); the same analyses at age 70 showed 4.49 (2.33 to 7.19) per 1000 person years higher incidence (1.65, 1.25 to 2.18).

**Conclusion:**

Multimorbidity, particularly when onset is in midlife rather than late life, has a robust association with subsequent dementia. The increasingly younger age at onset of multimorbidity makes prevention of multimorbidity in people with a first chronic disease important.

## Introduction

Increase in human life expectancy over the past two centuries implies that adults commonly live to age 65 and beyond, particularly in high income countries. A major consequence of the ageing of the population is the growing prevalence of dementia, which increases markedly after age 65.^1^ Alzheimer's disease, the primary cause of dementia, is a complex, multisystemic disease for which an effective cure remains elusive. As the pathophysiological process underlying Alzheimer's disease and related dementias unfolds over several decades,^2^ interest is increasing in how risk factors over the life course, including chronic diseases, shape the risk of dementia at older ages.^3^

A further consequence of population ageing is the increase in multimorbidity,^4^ ^5^ ^6^ conventionally defined as the presence of two or more chronic diseases irrespective of the severity of such conditions. Recent estimates suggest that more than 50% of older adults in high income countries report multiple chronic conditions, although multimorbidity is not confined to older adults.^4^ ^5^ The development of chronic diseases at younger ages has implications for their management, the risk of premature mortality, and the cost of care.^7^ Multimorbidity is estimated to have an adverse effect on patients’ outcomes and healthcare systems that is greater than that of chronic conditions considered indivdually.^8^

In older adults with dementia, the presence of several comorbid conditions is common.^9^ ^10^ A recent study of older adults (mean age 75 years) followed for a mean of 8.4 years reported higher risk of dementia in those with multimorbidity,^11^ but studies that have followed individuals for longer are lacking. Recent studies also suggest that the risk of dementia is higher in people with cardiometabolic disease in midlife rather than late life,^12^ ^13^ suggesting that age at onset of multimorbidity is an important determinant of risk of dementia. Accordingly, we examined whether longer duration of multimorbidity and severity of multimorbidity (defined as three or more chronic conditions), implying earlier age at onset of multimorbidity, increase the risk of dementia at older ages in a study spanning 30 years. The main focus was on any multimorbidity, but in exploratory analyses we also examined the importance of chronic disease dyads and their associations with dementia. Besides dementia, the primary outcome, we examined the role of age at onset of multimorbidity and subsequent mortality.

## Methods

The ongoing Whitehall II cohort study was established in 1985-88 among 10 308 employees (6895 men and 3413 women) of the British civil service, based in London at recruitment to the study and aged 35-55 years.^14^ Follow-up clinical examinations have taken place approximately every four to five years since baseline (1991-93, 1997-99, 2002-04, 2007-09, 2012-13, 2015-16, and an ongoing wave). Participants’ data were linked to UK National Health Service (NHS) electronic health records for all but 10 participants (99.9%). Most of the healthcare in the UK is provided by the NHS, including inpatient and outpatient care, and record linkage is possible owing to the unique NHS identifier held by all UK residents. Data from linked records were updated annually until 31 March 2019. Research ethics approvals and written informed consent from participants were renewed at each contact.

### Multimorbidity

We defined multimorbidity as the presence of at least two chronic conditions out of a predefined list of 13 chronic diseases, excluding dementia (the outcome in these analyses), that are prevalent in sufficient numbers across the adult life course. Multimorbidity should include at least 12 conditions,^15^ and we chose our list from previous research on multimorbidity.^4^ To examine severity of multimorbidity, we also re-categorised this measure as zero or one, two, and three or more chronic conditions. Ascertainment of these conditions was based on data from clinical examinations in the study as well as linkage to electronic health records using the NHS identification number. Electronic health records included the Hospital Episode Statistics (HES) database, the Mental Health Services Data Set, which in addition to inpatient and outpatient data also contains data on care in the community, and the national cancer registry.

The chronic diseases considered were coronary heart disease (ICD10 (international classification of diseases, 10th revision) codes: I20-I25, 12 lead resting electrocardiogram recording), stroke (ICD10: I60-I64, MONICA-Ausburg stroke questionnaire), heart failure (ICD10: I50), diabetes (ICD10: E10-E14, reported diabetes diagnosed by a doctor, use of diabetes drugs, or fasting glucose ≥7.0 mmol/L), hypertension (ICD10: I10-I16, systolic blood pressure ≥140 mm Hg, diastolic blood pressure ≥90 mm Hg, or use of antihypertensive drugs), cancer (malignant neoplasms ICD10: C00-C97), chronic kidney disease (ICD10: N18), chronic obstructive pulmonary disease (ICD10: J41-J44), liver disease (toxic liver disease, alcoholic liver disease, inflammatory liver disease, chronic hepatis, hepatic failure, fibrosis and cirrhosis, other diseases of liver, ICD10: K70-K74), depression (ICD10: F32-F33, use of antidepressants), mental disorders other than depression (ICD codes: F06, other mental disorders due to known physiological condition; F07: personality and behavioural disorders due to known physiological condition; F09: unspecified mental disorder due to known physiological condition; F20-F48 (excluding F32: depressive episode and F33: major depressive disorder, recurrent): mood/affective disorders, schizophrenia, schizotypal, delusional, and other non-mood psychotic disorders, anxiety, dissociative, stress related, somatoform and other nonpsychotic mental disorders; and F60-69 (excluding F65: paraphilias and F66: other sexual disorders): disorders of adult personality and behaviour), Parkinson’s disease (ICD10: G20), and arthritis/rheumatoid arthritis (ICD10: M15-M19, M05, M06).

### Primary outcome: dementia

Cases of dementia came from HES, the Mental Health Services Data Set, and the mortality register up to 31 March 2019. We used ICD-10 codes F00-F03, F05.1, G30, and G31 to identify all cause dementia. The sensitivity and specificity of dementia ascertainment based on HES data are 78.0% and 92.0%, respectively.^16^ Use of the Mental Health Services Data Set, a national database that includes information on dementia for people in contact with mental health services in hospitals, outpatient clinics, and the community, means that the sensitivity in our study is likely to be further improved.^17^ We set date of dementia at the first record of a diagnosis of dementia in any of the three databases.

### Secondary outcome: mortality

Death from any cause was the secondary outcome. We obtained mortality data until 31 March 2019 from the British national mortality register (NHS Central Registry) by using the NHS identification number of each participant.

### Covariates

Sociodemographic covariates included age, sex, ethnicity (white, non-white), education (lower secondary school or less, upper secondary school, university) and marital status (married/cohabiting, other). Health behaviours included as covariates reflected those in previous research on dementia,^18^ including time spent in moderate and vigorous physical activity, alcohol consumption (none in the previous week, 1-14 units/week, >14 units/week), smoking (never smoker, ex-smoker, current smoker), and frequency of fruit and vegetable consumption (less than daily, once a day, twice or more a day).

### Statistical analysis

We examined the association of individual chronic diseases and multimorbidity with incident dementia over the follow-up by using Cox proportional hazards regression with age as the time scale; all analyses excluded participants with prevalent dementia at the start of follow-up. Participants were censored at the date of record of dementia, death, or 31 March 2019, whichever came first. People who died during the follow-up were censored at date of death to account for the competing risk of death by using cause specific hazard models.^12^ ^19^ We plotted Schoenfeld residuals to verify the proportional hazards assumption. All analyses were first stratified on birth cohort (5 year groups) to take differences in baseline hazard due to birth year into account and adjusted for age (as time scale) and sex (model 1). They were then also adjusted for ethnicity, education, and marital status (model 2) and subsequently also for health behaviours (smoking status, alcohol consumption, fruit and vegetable consumption, and physical activity) (model 3).

We examined the overall association between each chronic disease (separate models) and incident dementia by entering chronic diseases and covariates as time varying variables, with follow-up beginning at study baseline (1985-88). Then, for each participant, we extracted disease status for all 13 chronic conditions and covariates at age 55, 60, 65, and 70 years (time invariant variable) and analysed these in separate models with follow-up for dementia beginning at age 55, 60, 65, and 70.

We then constructed a measure of multimorbidity status, defined as two or more chronic diseases, at 55, 60, 65, and 70 years old. Here, again, we used separate models with follow-up for dementia starting at age 55, 60, 65, and 70 to examine associations with incidence of dementia. For the analyses of multimorbidity at age 60, 65, and 70, we treated the age at onset of multimorbidity as a categorical variable in 5 year age bands to allow examination of the role of age at onset of multimorbidity on incident dementia over the follow-up. For example, for analysis of multimorbidity at age 65, we categorised the age at onset of multimorbidity as follows: no multimorbidity and multimorbidity before age 55, between ages 55 and 60, and between ages 60 and 65 years. Using these categories as a continuous variable, we used a test for trend to determine whether younger age at onset of multimorbidity was associated with an increased risk of dementia over the follow-up. For the analysis of multimorbidity status at age 70, we also used age at onset of multimorbidity as a continuous measure to determine the effect of every 5 year younger age at onset of multimorbidity on risk of dementia. We did further analyses using multimorbidity status and covariates treated as time varying variables to estimate the overall association between multimorbidity and incidence of dementia over the follow-up.

To examine the association of severity of multimorbidity and dementia, we re-categorised the multimorbidity measure as zero or one, two, and three or more chronic conditions. We examined the association of this measure at age 55, 60, 65, and 70 in separate models, first as a time invariant variable with subsequent dementia and then overall with this measure modelled as a time varying measure.

In exploratory analyses, we examined dyads of chronic conditions for their association with incidence of dementia over the follow-up in separate models, adjusted for covariates as in the main analyses and the presence of any disease other than those considered in the dyad. In additional analyses, we excluded Parkinson’s disease from the list of chronic conditions and reran the multimorbidity analyses using 12 chronic conditions to ensure that the results were not driven by possible Parkinson’s dementia.

In secondary analyses, we used the methods described above with mortality as the outcome. We used two sided P values with the threshold for statistical significance at α=0.05. Methods used in the analysis of incident dementia are summarised in supplementary table A. We used R version 4.0.3 (R Core Team) for all analyses.

### Patient and public involvement

Participants of the Whitehall II study were not involved in setting the research question or the outcome measures, nor were they involved in developing plans for recruitment, design, or implementation of the study. No participants were asked for advice on interpretation or writing up of results. We recognise that public involvement has great value and contributes to improving the quality of research, but we unfortunately did not have the necessary funding to involve patients. The Whitehall study investigators are thinking of new solutions to better involve the public in the future. However, we sought suggestions from a patient reviewer during the revision process of the article, and these helped to improve the clarity of the paper. Finally, all results are disseminated to study participants via newsletters and a website, which has a participant portal (https://www.ucl.ac.uk/epidemiology-health-care/research/epidemiology-and-public-health/research/whitehall-ii/participants-area) and to a larger audience via media outreach.

## Results

After excluding participants without a follow-up and missing covariates data, we included 10 095 of the 10 308 participants at baseline in 1985 for analysis of multimorbidity as a time varying measure (flowchart in supplementary figure A**)**. The flowchart also shows the selection of the analytical sample at 55, 60, 65, and 70 years old. The primary reason for missing data in these analyses was death before reaching target age; in the analysis of multimorbidity at age 70, it was death (n=904) or participants not yet being 70 years at the date of censoring (n=1311) (supplementary figure A). Supplementary table B shows characteristics of the 203 participants excluded owing to missing covariates.

**Fig 1:**
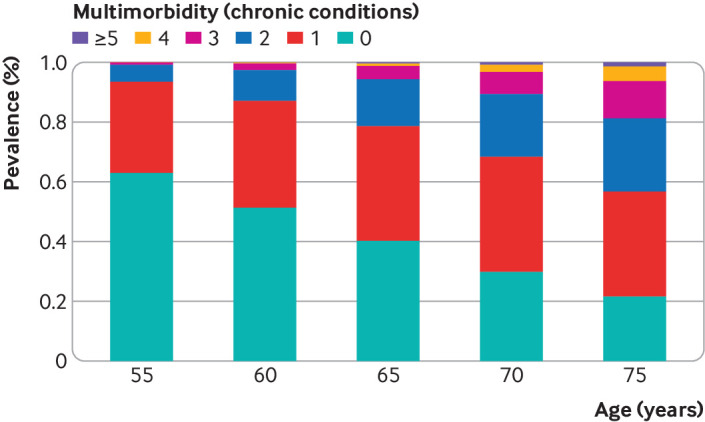
Prevalence of multiple chronic diseases at age 55, 60, 65, and 70 years. Chronic conditions were coronary heart disease, stroke, heart failure, diabetes, hypertension, cancer, chronic kidney disease, chronic obstructive pulmonary disease, liver disease, depression, mental disorders, Parkinson’s disease, and arthritis/rheumatoid arthritis

Table 1 shows the baseline characteristics of the 10 095 participants according to dementia status at the end of the follow-up. Over a median follow-up of 31.7 (interquartile range 31.1-32.6) years, a total of 639 cases of dementia were ascertained. The most frequent chronic condition among people with dementia was hypertension, followed by coronary heart disease, depression, and diabetes. The prevalence of all individual chronic conditions increased with age (supplementary figure B), with some chronic conditions showing a steady increase (for example, diabetes) and others a more rapid increase between age 65 and 70 (for example, chronic obstructive pulmonary disease, chronic kidney disease). At age 55, 63.0% (6259/9937) of participants had none of the 13 chronic diseases considered compared with 29.8% (2322/7783) at age 70 (fig 1); 6.6% (655/9937) of participants at age 55 reported multimorbidity (two or more chronic conditions), and 31.7% (2464/7783) reported it at age 70.

**Table 1:** Characteristics of participants by dementia status at end of follow-up. Values are numbers (percentages) unless stated otherwise

Characteristics at baseline (1985-88)	Dementia status at March 2019	
	Dementia* (n=639)	No dementia (n=9456)
**Demographics**		
Male sex	374 (58.5)	6423 (67.9)
Mean (SD) age, years	49.9 (4.9)	44.6 (6.0)
Education:		
Low secondary school or lower	378 (59.2)	4397 (46.5)
High school diploma	135 (21.1)	2551 (27.0)
University degree or higher	126 (19.7)	2508 (26.5)
Ethnicity:		
White	547 (85.6)	8545 (90.4)
Non-white	92 (14.4)	911 (9.6)
Marital status:		
Married/cohabiting	452 (70.7)	7032 (74.4)
Single/divorced/widowed	187 (29.3)	2424 (25.6)
**Health behaviours**		
Smoking:		
Never smoker	314 (49.1)	4707 (49.8)
Former smoker	197 (30.8)	3049 (32.2)
Current smoker	128 (20.0)	1700 (18.0)
Mean (SD) hours of MVPA per week	3.4 (3.7)	3.9 (4.3)
Alcohol consumption:		
0 units/week	158 (24.7)	1676 (17.7)
1-14 units/week	352 (55.1)	5506 (58.2)
>14 units/week	129 (20.2)	2274 (24.0)
Fruit/vegetable consumption:		
Once/day	275 (43.0)	3937 (41.6)
≥Once/day	364 (57.0)	5519 (58.4)
**Chronic conditions at end of follow-up** *		
Coronary heart disease	177 (27.7)	1996 (21.1)
Stroke	66 (10.3)	388 (4.1)
Heart failure	62 (9.7)	529 (5.6)
Diabetes	158 (24.7)	1560 (16.5)
Hypertension	498 (77.9)	6140 (64.9)
Cancer	103 (16.1)	2113 (22.3)
Chronic kidney disease	65 (10.2)	476 (5.0)
Chronic obstructive pulmonary disease	62 (9.7)	474 (5.0)
Liver disease	17 (2.7)	268 (2.8)
Depression	174 (27.2)	1090 (11.5)
Mental disorders	100 (15.6)	418 (4.4)
Parkinson’s disease	41 (6.4)	104 (1.1)
Arthritis/rheumatoid arthritis	140 (21.9)	1453 (15.4)
**Multimorbidity status at end of follow-up** **†**		
0 or 1 chronic condition	189 (29.6)	4645 (49.1)
Multimorbidity (≥2 chronic conditions)	450 (70.4)	4811 (50.9)

MVPA=moderate and vigorous physical activity; SD=standard deviation.

* Of 639 cases of dementia, 274 were due to Alzheimer’s disease, 106 were vascular dementia, 29 were due to Parkinson’s disease, 100 were due to other causes, and for 130 cases cause was unspecified/missing.

† End of follow-up defined as date of diagnosis of dementia, date of death, or March 2019, whichever came first.

In analyses of individual chronic diseases (table 2), cancer (hazard ratio 1.06, 95% confidence interval 0.85 to 1.31) was not associated with dementia when treated as time varying variables or in the age specific analyses. Apart from Parkinson’s disease, mental disorders had the highest hazard ratio (13.51, 6.53 to 27.95) at age 60, with a progressive decrease in the association with age such that prevalent mental disorders at age 70 had a hazard ratio for dementia of 2.05 (0.97 to 4.35). Although small numbers did not allow analyses at ages below 70, Parkinson’s disease had the highest hazard ratio in the time varying analyses (8.16, 5.68 to 11.73). The association of dementia with stroke, heart failure, diabetes, chronic obstructive pulmonary disease, and mental disorders weakened when these chronic conditions were measured at older ages, whereas that with depression showed the opposite pattern (table 2).

**Table 2:** Association between chronic diseases considered individually and risk of subsequent dementia. Values are hazard ratios (95% confidence intervals)

Chronic disease	Prevalent at 55 years* †	Prevalent at 60 years* †	Prevalent at 65 years* †	Prevalent at 70 years* †	Overall† ‡
Coronary heart disease	1.35 (0.94 to 1.95)	1.46 (1.11 to 1.92)	1.31 (1.03 to 1.66)	1.16 (0.93 to 1.45)	1.25 (1.04 to 1.50)
Stroke	NA	3.55 (1.46 to 8.59)	2.68 (1.38 to 5.20)	1.62 (0.89 to 2.95)	3.37 (2.54 to 4.49)
Heart failure	NA	NA	3.44 (1.41 to 8.35)	1.39 (0.69 to 2.81)	2.49 (1.88 to 3.32)
Diabetes	2.31 (1.56 to 3.43)	2.21 (1.63 to 2.99)	1.98 (1.54 to 2.55)	1.60 (1.25 to 2.05)	1.63 (1.35 to 1.97)
Hypertension	1.37 (1.15 to 1.62)	1.29 (1.10 to 1.51)	1.29 (1.10 to 1.52)	1.31 (1.10 to 1.56)	1.55 (1.27 to 1.88)
Cancer	0.97 (0.50 to 1.88)	0.99 (0.60 to 1.64)	0.69 (0.44 to 1.09)	0.88 (0.63 to 1.24)	1.06 (0.85 to 1.31)
Chronic kidney disease	NA	NA	NA	5.18 (2.29 to 11.70)	2.98 (2.22 to 3.99)
Chronic obstructive pulmonary disease	NA	NA	3.64 (1.49 to 8.90)	1.12 (0.46 to 2.72)	2.20 (1.63 to 2.97)
Liver disease	NA	NA	NA	NA	1.89 (1.11 to 3.21)
Depression	1.42 (0.94 to 2.15)	1.71 (1.25 to 2.35)	1.79 (1.36 to 2.35)	1.93 (1.50 to 2.50)	2.95 (2.44 to 3.57)
Mental disorders	NA	13.51 (6.53 to 27.95)	6.66 (3.61 to 12.27)	2.05 (0.97 to 4.35)	5.73 (4.48 to 7.34)
Parkinson’s disease	NA	NA	21.24 (8.66 to 52.09)	19.31 (8.45 to 44.11)	8.16 (5.68 to 11.73)
Arthritis/Rheumatoid arthritis	NA	1.66 (0.68 to 4.05)	1.51 (0.91 to 2.51)	1.14 (0.78 to 1.67)	1.49 (1.22 to 1.81)

NA=not applicable (insufficient number of cases (<5) to allow analysis).

* Four separate analyses were run for prevalent disease at age 55, 60, 65, and 70 years, with median follow-up of 19.6, 14.8, 10.1, and 6.7 years, respectively. All chronic diseases were studied in separate models.

† Analyses were stratified on birth cohort (5 year groups) and adjusted for age (as time scale), sex, ethnicity, education, marital status, and health behaviours (smoking, alcohol consumption, physical activity, and diet). Covariate measurement was concurrent with measure of chronic diseases.

‡ All chronic diseases were studied in separate models, and covariates were entered as time varying measures. Median follow-up was 31.7 years.

Compared with people without any of the 13 chronic conditions, multimorbidity (two or more of 13 chronic conditions) at age 55 was associated with a higher incidence rate (difference 1.56 (95% confidence interval 0.62 to 2.77) per 1000 person years) and a hazard ratio of subsequent dementia of 2.44 (1.82 to 3.26) in analyses that included adjustment for all covariates (table 3). When we considered multimorbidity status at age 60, 65, and 70, younger age at onset of multimorbidity was associated with higher risk of dementia (all P for trend <0.001). For example, at age 65 years, participants with onset of multimorbidity before age 55 (corresponding to duration of multimorbidity of >10 years) had a hazard ratio of subsequent dementia of 2.46 (1.80 to 3.36; difference in incidence per 1000 person years 3.86, 1.80 to 6.52) compared with 1.51 (1.16 to 1.97; difference in incidence per 1000 person years 1.85, 0.64 to 3.39) in those with onset of multimorbidity between age 60 and 65. In the analysis on multimorbidity status at age 70, a 5 year younger age at onset of multimorbidity was associated with a hazard ratio for dementia of 1.18 (1.04 to 1.34; data not tabulated). When we examined multimorbidity as a time varying measure (table 3), the difference in incidence of dementia per 1000 person years between participants with and without multimorbidity was 1.59 (1.51 to 1.67) and the fully adjusted hazard ratio was 2.36 (1.97 to 2.81). Removing Parkinson’s disease from the list of chronic conditions used to construct the multimorbidity measure did not affect the findings (supplementary table C); for the time varying multimorbidity measure, the difference in incidence per 1000 person years was 1.55 (1.47 to 1.63) and the corresponding hazard ratio for dementia was 2.29 (1.92 to 2.73).

**Table 3:** Association between multimorbidity (≥2 chronic conditions) and subsequent risk of dementia as a function of age at onset of multimorbidity*

Multimorbidity status		No of dementia cases/total No		Incidence rate per 1000 person years		Incidence rate difference (95% CI) per 1000 person years		Hazard ratio (95% CI)				
								Model 1†		Model 2†		Model 3†
**At age 55 years (median follow-up 19.6 (IQR 15.6-25.1) years)**												
0 or 1 chronic condition	587/9282		3.10		0 (reference)		1 (reference)		1 (reference)		1 (reference)	
Multimorbidity	51/655		4.67		1.56 (0.62 to 2.77)		2.51 (1.87 to 3.35)		2.47 (1.84 to 3.30)		2.44 (1.82 to 3.26)	
**At age 60 years (median follow-up 14.8 (IQR 10.8-20.2) years)**												
0 or 1 chronic condition	542/8497		4.04		0 (reference)		1 (reference)		1 (reference)		1 (reference)	
Multimorbidity between 55 and 60	41/642		4.64		0.60 (−0.38 to 1.90)		1.45 (1.05 to 1.99)		1.40 (1.02 to 1.93)		1.39 (1.01 to 1.91)	
Multimorbidity before age 55	50/621		6.46		2.42 (1.09 to 4.12)		2.57 (1.91 to 3.45)		2.52 (1.88 to 3.39)		2.46 (1.83 to 3.31)	
P for trend	-		-		-		<0.001		<0.001		<0.001	
**At age 65 years (median follow-up 10.1 (IQR 6.1-15.4) years)**												
0 or 1 chronic condition	467/7444		5.59		0 (reference)		1 (reference)		1 (reference)		1 (reference)	
Multimorbidity between 60 and 65	64/858		7.44		1.85 (0.64 to 3.39)		1.55 (1.19 to 2.01)		1.53 (1.17 to 1.99)		1.51 (1.16 to 1.97)	
Multimorbidity between 55 and 60	39/602		6.83		1.24 (−0.24 to 3.22)		1.49 (1.08 to 2.07)		1.44 (1.03 to 2.00)		1.41 (1.01 to 1.96)	
Multimorbidity before age 55	45/578		9.45		3.86 (1.80 to 6.52)		2.57 (1.88 to 3.50)		2.52 (1.85 to 3.44)		2.46 (1.80 to 3.36)	
P for trend	-		-		-		<0.001		<0.001		<0.001	
**At age 70 years (median follow-up 6.7 (IQR 3.0- 11.4) years)**												
0 or 1 chronic condition	352/5319		8.44		0 (reference)		1 (reference)		1 (reference)		1 (reference)	
Multimorbidity between 65 and 70	71/894		10.78		2.34 (0.84 to 4.23)		1.33 (1.03 to 1.72)		1.32 (1.02 to 1.70)		1.29 (1.001 to 1.67)	
Multimorbidity between 60 and 65	50/683		10.76		2.32 (0.41 to 4.82)		1.45 (1.07 to 1.95)		1.41 (1.04 to 1.89)		1.38 (1.02 to 1.86)	
Multimorbidity between 55 and 60	32/470		10.96		2.52 (−0.09 to 6.10)		1.50 (1.04 to 2.15)		1.42 (0.98 to 2.04)		1.36 (0.94 to 1.96)	
Multimorbidity before age 55	37/417		17.38		8.94 (4.65 to 14.58)		2.80 (1.99 to 3.94)		2.71 (1.92 to 3.83)		2.60 (1.84 to 3.67)	
P for trend	-		-		-		<0.001		<0.001		<0.001	
**Overall (multimorbidity and covariates as time varying measures; median follow-up 31.7 (IQR 31.1-32.6) years)**												
0 or 1 chronic condition	189/4834		1.30		0 (reference)		1 (reference)		1 (reference)		1 (reference)	
Multimorbidity	450/5261		2.89		1.59 (1.51 to 1.67)		2.44 (2.04 to 2.91)		2.44 (2.04 to 2.91)		2.36 (1.97 to 2.81)	

IQR=interquartile range.

* Multimorbidity defined as ≥2 chronic conditions out of 13 conditions: coronary heart disease, stroke, heart failure, diabetes, hypertension, cancer, chronic kidney disease, chronic obstructive pulmonary disease, liver disease, depression, mental disorders, Parkinson’s disease, and arthritis/rheumatoid arthritis.

† Model 1: stratified on birth cohort (5 year groups) and adjusted for age (as time scale) and sex; model 2: model 1+ethnicity, education, and marital status; model 3: model 2+health behaviours (smoking, alcohol consumption, physical activity, and diet). Covariate measurement was concurrent with measure of multimorbidity

**Fig 2:**
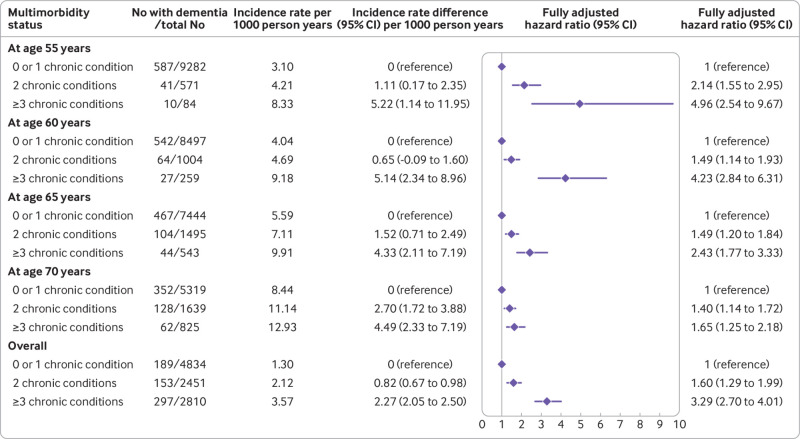
Association between number of chronic conditions (≤1, 2, and ≥3) and subsequent risk of dementia. Chronic conditions considered were coronary heart disease, stroke, heart failure, diabetes, hypertension, cancer, chronic kidney disease, chronic obstructive pulmonary disease, liver disease, depression, mental disorders, Parkinson’s disease, and arthritis/rheumatoid arthritis. Analyses were stratified on birth cohort (5 year groups) and adjusted for age (as time scale), sex, ethnicity, education, marital status, and health behaviours (smoking, alcohol consumption, physical activity, and diet). Covariates measurement was concurrent with measure of multimorbidity

Re-categorisation of multimorbidity according to severity (≤1, 2, ≥3 of 13 chronic conditions; fig 2) showed that participants with three or more chronic conditions at age 55 had a hazard ratio for dementia of 4.96 (2.54 to 9.67) compared with those with no or one chronic condition at age 55; the difference in incidence per 1000 person years was 5.22 (1.14 to 11.95). We observed a pattern of progressive weakening of this association when onset of multimorbidity was at older ages. When analysed as a time varying variable (fig 2), compared with participants with no or one chronic disease, those with two chronic diseases had a difference in incidence per 1000 person years of 0.82 (0.67 to 0.98) and a hazard ratio for dementia of 1.60 (1.29 to 1.99) and those with three or more chronic conditions had a difference in incidence per 1000 person years of 2.27 (2.05 to 2.50) and a hazard ratio of 3.29 (2.70 to 4.01).

**Fig 3:**
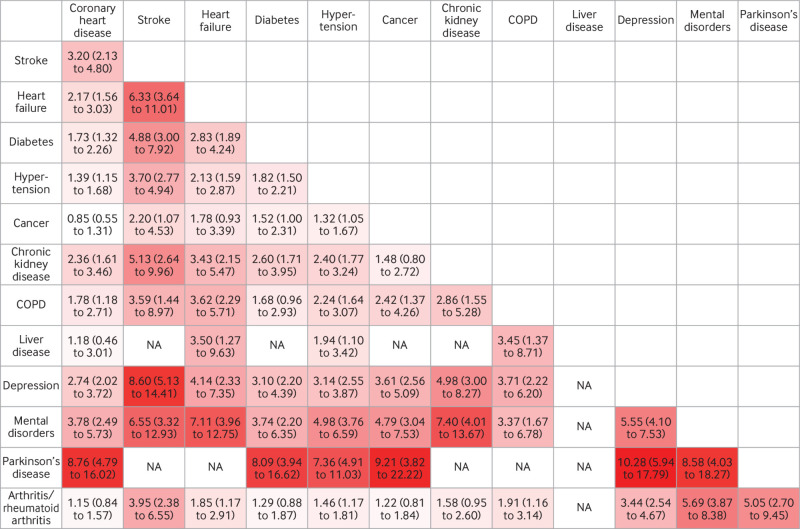
Hazard ratios (95% confidence intervals) for association between dyads of chronic diseases and subsequent risk of dementia. Analyses were stratified on birth cohort (5 year groups) and adjusted for age (as time scale), sex, ethnicity, education, marital status, and health behaviours (smoking, alcohol consumption, physical activity, and diet), as well as presence of any other chronic disease not included in dyad; measurements were concurrent with measure of multimorbidity. Colour gradient is used to reflect strength of associations, from white (lowest hazard ratio) to red (highest hazard ratio). COPD=chronic obstructive pulmonary disease; NA=not applicable (insufficient number of cases (<5) to allow analysis)

Figure 3 shows exploratory analyses of association of dyads of chronic diseases, treated as time varying covariates, with subsequent dementia; the number of cases of dementia and total numbers in analyses for the dyads in question are shown in supplementary figure C. The dyads most strongly associated with the risk of dementia were those involving Parkinson’s disease, particularly Parkinson’s disease combined with depression (hazard ratio 10.28, 5.94 to 17.79), cancer (9.21, 3.82 to 22.22), coronary heart disease (8.76, 4.79 to 16.02), mental disorders (8.58, 4.03 to 18.27), diabetes (8.09, 3.94 to 16.62), and hypertension (7.36, 4.91 to 11.03). Other pairs of chronic diseases strongly associated with the risk of subsequent dementia involved depression and stroke (hazard ratio 8.60, 5.13 to 14.41) and mental disorders and chronic kidney disease (7.40, 4.01 to 13.67), heart failure (7.11, 3.96 to 12.75), or stroke (6.55, 3.32 to 12.93). Heart failure combined with stroke also had a strong association with the risk of subsequent dementia (hazard ratio 6.33, 3.64 to 11.01).

The main analyses on multimorbidity were repeated for mortality. A total of 2136 participants died over a median follow-up of 31.7 (interquartile range 31.1-32.7) years. At the end of follow-up, the prevalence of all chronic diseases except arthritis/rheumatoid arthritis was higher in participants who died (supplementary table D). When studied separately as time varying variables, all chronic conditions were associated with mortality (supplementary table E); cancer was the chronic disease with the highest hazard ratio for mortality (9.03, 8.06 to 10.13).

Multimorbidity (at least two chronic conditions) at age 55, 60, 65, and 70 years was associated with mortality (supplementary table F), with the hazard ratios being higher for younger age at onset of multimorbidity (P for tend <0.001 for multimorbidity at age 60, 65, and 70). In analyses using time varying multimorbidity, the hazard ratio for mortality was 4.86 (4.36 to 5.41) and decreased to 3.13 (3.01 to 3.65) after cancer was removed from the list of chronic conditions. Analysis of severity of multimorbidity (≤1, 2, ≥3 chronic conditions) without cancer showed that compared with participants with no or one chronic condition, those with two (hazard ratio 1.73, 1.52 to 1.98) and three or more chronic conditions (5.09, 4.47 to 5.78) had a higher risk of subsequent mortality (supplementary table G).

## Discussion

In this analysis of more than 10 000 individuals followed for more than 30 years, multimorbidity (two or more chronic conditions) was associated with a higher risk of subsequent dementia. This was the case with multimorbity at age 55, 60, 65, and 70 years; the strongest associations were seen in those with multimorbidity at age 55, with a weakening of associations for onset of multimorbidity at older ages. When multimorbidity was defined as three or more chronic conditions, the importance of younger age of onset of multimorbidity for the risk of dementia was further accentuated. Compared with people with no or one chronic condition, those with three or more chronic conditions at age 55 had a nearly fivefold higher risk of dementia; the risk was 1.7-fold higher when onset of multimorbidity was at age 70. To our knowledge, this study is the first to show that multimorbidity in midlife is associated with higher risk of dementia at older ages.

### Comparison with other studies

The evidence on the association between multimorbidity and dementia is mostly based on cross sectional studies,^9^ ^10^ ^20^ ^21^ in which multimorbidity rates have been shown to be systematically higher in people with dementia. We found only two prospective studies on the association between multimorbidity and dementia. A recent study of 2478 older adults (mean age at multimorbidity assessment 75 years) with a mean follow-up of 8.4 years found neuropsychiatric, cardiovascular, and sensory impairment/cancer multimorbidity to be associated with dementia.^11^ Another study in 2176 adults (mean age at multimorbidity assessment 78.5 years, median follow-up 4 years) reported an association between multimorbidity and a combined outcome comprised of mild cognitive impairment and dementia.^22^

Previous studies have shown multimorbidity to be associated with mild cognitive impairment,^23^ decline in cognitive function,^24^ ^25^ ^26^ and biomarkers of neurodegeneration.^27^ However, these studies were either cross sectional or based on older adults with an age at assessment of multimorbidity between 65 and 74 years.^23^ ^24^ ^25^ ^26^ ^27^ One longitudinal study using a combined outcome of mild cognitive impairment and dementia and one cross sectional study on mild cognitive impairment also reported stronger associations with severity of multimorbidity.^22^ ^23^ The only study to have examined the role of age at onset of multimorbidity is a cross sectional study with data from low and middle income countries, which found the association between multimorbidity and mild cognitive impairment to be similar in the group with an age range of 50-64 years and in those who were 65 years and older.^23^ We were able to examine multimorbidity, assessed between age 55 and 70, and subsequent dementia to show the importance of both age at onset and severity (reflected in number of chronic conditions) of multimorbidity in shaping risk of dementia.

Studies show associations between several individual chronic conditions and subsequent dementia; these are likely to involve a range of mechanisms.^28^ Cardiometabolic diseases in midlife rather than at older ages are thought to increase the risk of dementia.^12^ ^13^ For depression, some studies suggest that the association is confined to depression in old age, which may be a marker of preclinical dementia.^29^ ^30^ Mental disorders, particularly when onset is at younger ages, have been shown to be associated with increased risk of dementia at older ages, notably in analyses of disease trajectories.^31^ ^32^ ^33^ ^34^ ^35^ The results on liver diseases are inconsistent,^36^ ^37^ ^38^ cancer has a “protective” association with dementia,^39^ and the role of musculoskeletal diseases remains unclear. Parkinson’s disease has a strong association with dementia,^40^ ^41^ ^42^ as can be seen in our analyses of chronic diseases considered individually or in dyads. However, removal of Parkinson’s disease did not substantially modify the association between multimorbidity and subsequent dementia or the results in relation to the importance of younger age at onset of multimorbidity in increasing the risk of dementia at older ages. We retained all 13 chronic conditions in the analyses rather than only those found to be associated with dementia, as this approach allows the results to be generalisable to multimorbidity, rather than specific chronic conditions. This is particularly true for the findings on age at onset and severity of multimorbidity, although the precise estimate of the association between multimorbidity and dementia is likely to be dependent on the type and number of chronic conditions considered in the analysis.

Although chronic conditions considered individually affect the risk of dementia, the research on multimorbidity shows specific cumulative effects of clustering of chronic diseases, accelerating cognitive decline and increasing the risk of dementia.^22^ ^43^ In our study, the analyses of severity of multimorbidity suggest that the cumulative effect of diseases rather than specific combinations drive the association with dementia. Inflammatory processes may play a role, as inflammation is a risk factor for many chronic conditions and dementia.^11^ ^44^ ^45^ Interactions between and/or accumulation of drugs prescribed to treat individual chronic conditions have also been suggested to affect cognitive ageing and the incidence of dementia.^22^

### Strengths and limitations of study

Strengths of this study include use of data from a longitudinal cohort study with a median follow-up of 31.7 years to study the role of multimorbidity in midlife and late life for incident dementia. To our knowledge, this study is the first to use a longitudinal framework to examine the association of multimorbidity with the risk of dementia on such a large scale. Three aspects of the analytical strategy are notable: the focus on age at onset of multimorbidity along with analysis of multimorbidity as a time varying measure to examine the role of age at onset; consideration of the severity of multimorbidity (two and three conditions), showing the effect of clustering of chronic diseases when the number of diseases is greater; and use of mortality as a secondary outcome for which results are similar to those in the literature, increasing confidence in our findings on dementia.

This study has some limitations. Ascertainment of dementia was based on linkage to electronic health records. Although this approach is not “gold standard,” it has the advantage of dementia status being available for everyone rather than only those participants who agree to a face to face assessment over the course of the study. The limitation of this approach is that it may lead to misclassification of some cases of dementia, particularly milder cases,^16^ and the cause of dementia is not fully described leading to analyses only on all cause dementia. Another limitation is that the Whitehall II study is based on participants who were all in employment at recruitment and are likely to be healthier than the general population, in terms of both incidence of disease and risk factor profiles. Nevertheless, this does not necessarily affect the association between risk factors and the disease of interest.^46^ A previous study reported that the association between risk of cardiovascular disease and cardiovascular risk factors in the Whitehall II study was similar to that in general population studies.^47^ A further limitation is lack of statistical power to examine dyads according to age at onset of pairs of chronic diseases to allow monitoring of specific chronic conditions. However, the focus of these analyses was multimorbidity rather than specific dyads, and our results on Parkinson’s disease show the association of multimorbidity with subsequent dementia to be independent of specific diseases. Finally, these data are not suited to studying the role of severity of individual chronic conditions or the role played by drug interactions; these aspects might improve understanding of the manner in which multimorbidity at all ages shapes the risk of dementia at older ages.

### Conclusions

Given the lack of effective treatment for dementia and its personal and societal implications, finding targets for prevention of dementia is imperative. Multimorbidity is increasingly prevalent, starting in early adulthood and midlife. Our results show that multimorbidity is associated with an increased risk of dementia at older ages, even more so when onset of multimorbidity is in midlife rather than late life. These findings highlight the role of prevention and management of chronic diseases over the course of adulthood to mitigate adverse outcomes in old age. Multimorbidity is already known to affect use of healthcare services, quality of life, and risk of mortality; our study adds dementia to that list.

## What is already known on this topic

Robust evidence shows that multimorbidity is highly prevalent, particularly at older ages and in people living with dementia
Most studies on the association between multimorbidity and dementia are cross sectional, and the few prospective studies have a short follow-up with multimorbidity measured only at older ages
Studies that examine whether earlier age at onset of multimorbidity affects the risk of subsequent dementia are lacking

## What the study adds

Multimorbidity, defined as co-occurrence of two or more chronic conditions, was associated with a 2.4-fold increase in risk of dementia over a median follow-up of 32 years
For every 5 year younger age at onset of multimorbidity up to age 70, the risk of dementia was higher by 18%
Increased severity of multimorbidity strengthened associations with dementia, particularly multimorbidity in midlife

## Data Availability

Whitehall II data cannot be shared publicly because of constraints dictated by the study’s ethics approval and institutional review board restrictions. The Whitehall II data are available for sharing within the scientific community. Researchers can apply for data access at https://www.ucl.ac.uk/epidemiology-health-care/research/epidemiology-and-public-health/research/whitehall-ii/data-sharing.

## Web extra

Extra material supplied by authors

Web appendix: Supplementary materials

## Notes

Contributors: CBH, SS, and ASM generated the hypothesis and designed the study. CBH, AF, SS, and ASM developed the study methods. AF and CBH curated the data. CBH, AF, and SS did the formal analyses. CBH, AF, SS, and ASM visualised and interpreted the data. CBH and ASM wrote the original draft of the manuscript. All authors discussed results and commented on the manuscript. SS and ASM supervised the project. ASM acquired funding. All authors had full access to all the data in the study and accept responsibility to submit for publication. CBH is the guarantor. The corresponding author attests that all listed authors meet authorship criteria and that no others meeting the criteria have been omitted.

The lead author (the manuscript’s guarantor) affirms that this manuscript is an honest, accurate, and transparent account of the study being reported; that no important aspects of the study have been omitted; and that any discrepancies from the study as planned (and, if relevant, registered) have been explained.

Dissemination to participants and related patient and public communities: All results are disseminated to study participants via newsletters and a website, which has a participant portal (https://www.ucl.ac.uk/epidemiology-health-care/research/epidemiology-and-public-health/research/whitehall-ii/participants-area ), and to a larger audience via media outreach.

Provenance and peer review: Not commissioned; externally peer reviewed.
