# Supplementary material for: Association between age at onset of multimorbidity and incidence of dementia: 30 year follow-up in Whitehall II prospective cohort study
Source: BMJ. 2022 Feb 2;376:e068005. doi: 10.1136/bmj-2021-068005 (PMC9086721; doi:10.1136/bmj-2021-068005)
Supplement: Supplementary file 1 — Web appendix: Supplementary materials [file benc068005.ww1.pdf]

Association between age at onset of multimorbidity and incidence of dementia: a 30-year follow-up in the Whitehall II prospective cohort study

Céline Ben Hassen[1], Aurore Fayosse[1], Benjamin Landré[1], Martina Raggi[1], Mikaela Bloomberg[1,2], Séverine Sabia[1,2], Archana Singh-Manoux[1,2]

[1]Université de Paris, Inserm U1153, Epidemiology of Ageing and Neurodegenerative diseases, Paris, France

[2]Department of Epidemiology and Public Health, University College London, UK

Supplemental data

Supplementary Table A. Tabular description of statistical models and methods for analysis of incidence of dementia.

Supplementary Table B. Baseline (1985-1998) characteristics of participants excluded and included in the analysis.

Supplementary Table C. Association between multimorbidity (≥ 2 chronic conditions) and subsequent risk of dementia as a function of age at onset of multimorbidity, excluding Parkinson's disease from the conditions considered in the definition of multimorbidity.

Supplementary Table D. Characteristics of participants as a function of mortality status at end of follow-up.

Supplementary Table E. Association between chronic diseases considered individually and subsequent risk of mortality.

Supplementary Table F. Association between multimorbidity (≥ 2 chronic conditions) as a function of age at onset of multimorbidity and subsequent risk of mortality.

Supplementary Table G. Association between number of chronic conditions (≤1, 2, and ≥3) and subsequent risk of mortality.

Supplementary Figure A. Flow-chart.

Supplementary Figure B. Prevalence of individual chronic diseases at age 55, 60, 65, and 70 years.

Supplementary Figure C. Number of dementia cases and total number of participants with the corresponding dyads

Supplementary Table A. Tabular description of statistical models and methods for analysis of incidence of dementia

Table/Figure number	Exposure	Model	Methods	Covariates	Outcome
**Table 2**<br>13 chronic diseases considered individually	Chronic conditions<br>Prevalent at age 55, 60, 65, 70 years	Fully-adjusted analysis for each chronic condition, age specific analyses	Cause-specific Cox proportional-hazards regression<br>Start of follow-up at age 55, 60, 65, 70 years, respectively	Age (time-scale), sex, ethnicity, education, marital status, and health behaviours, birth-cohort (5-year groups, stratification)<br>Covariates measured at the same age as chronic diseases	Incidence of Dementia
	Time-varying chronic conditions<br>(overall)	A fully-adjusted model for each chronic condition	Cause-specific time-varying Cox proportional-hazards regression<br>Start of follow-up at baseline	Age (time-scale), sex, ethnicity, education, marital status, and health behaviours, birth-cohort (5-year groups, stratification)<br>Covariates time-varying	Incidence of Dementia
**Table 3**<br>Multimorbidity<br>($\geq$ 2 chronic conditions)	Prevalence and age at onset of multimorbidity: at age 55, 60 (2 groups: onset 55-60, or <55 years), 65 (3 groups: onset 60-65, 55-60, or <55 years) and 70 years (4 groups: onset 65-70, 60-65, 55-60, or <55 years)	Model 1<br>age specific analysis	Cause-specific Cox proportional-hazards regression<br>Start of follow-up at age 55, 60, 65 and 70 years respectively	Model 1: age (time scale), sex, birth-cohort (5-year groups, stratification)<br>Covariates measured at the same age as multimorbidity status	Incidence of Dementia
		Model 2<br>age specific analysis		Model 1 + ethnicity, education, marital status<br>Covariates measured at the same age as multimorbidity status	
		Model 3<br>age specific analysis		Model 2 + health behaviours<br>Covariates measured at the same age as multimorbidity status	
	Time-varying multimorbidity<br>(overall)	Model 1	Cause-specific time-varying Cox proportional-hazards regression<br>Start of follow-up at baseline	Model 1: age (time scale), sex, birth-cohort (5-year groups, stratification)<br>Covariates time-varying	Incidence of Dementia
		Model 2		Model 1 + ethnicity, education, marital status<br>Covariates time-varying	
		Model 3		Model 2 + health behaviours<br>Covariates time-varying	
**Figure 2**<br>Severity of Multimorbidity<br>(0-1, 2, or $\geq$3 chronic conditions)	Number of chronic conditions ($\leq$1, 2, and $\geq$3) at age 55, 60, 65, 70 years	Model 1<br>age specific analysis	Cause-specific Cox proportional-hazards regression<br>Start of follow-up at age 55, 60, 65, 70 years, respectively	Model 1: age (time scale), sex, birth-cohort (5-year groups, stratification)<br>Covariates measured at the same age as severity of multimorbidity	Incidence of Dementia
		Model 2<br>age specific analysis		Model 1 + ethnicity, education, marital status<br>Covariates measured at the same age as severity of multimorbidity	
		Model 3<br>age specific analysis		Model 2 + health behaviours<br>Covariates measured at the same age as severity of multimorbidity	
	Time-varying number of chronic conditions<br>(overall)	Model 1	Cause-specific time-varying Cox proportional-hazards regression<br>Start of follow-up at baseline	Model 1: age (time scale), sex, birth-cohort (5-year groups, stratification)<br>Covariates time-varying	Incidence of Dementia
		Model 2		Model 1 + ethnicity, education, marital status<br>Covariates time-varying	
		Model 3		Model 2 + health behaviours<br>Covariates time-varying	
**Figure 3**<br>Dyads of chronic conditions	Pairs of chronic conditions, out of 13<br>(overall)	A fully-adjusted model for each dyad of chronic conditions	Cause-specific time-varying Cox proportional-hazards regression<br>Start of follow-up at baseline	Age (time scale), sex, birth-cohort (5-year groups, stratification), ethnicity, education, marital status, health behaviours, prevalent other chronic disease not included in the dyad<br>Covariates time-varying	Incidence of Dementia

Supplementary Table B. Baseline (1985-1998) characteristics of participants excluded and included in the analysis

**Participant characteristics at baseline**		**Excluded from the analyses**	**Included in the analyses**
**N**		**N= 203**	**N = 10,095**
Sex	Men	95 (46.8)	6,797 (67.3)
	Women	108 (53.2)	3,298 (32.7)
Mean (SD) age, years		46.4 (6.3)	44.9 (6.0)
Education	Low secondary school or lower	119 (58.6)	4,775 (47.3)
	High school diploma	57 (28.1)	2,686 (26.6)
	University degree or higher	27 (13.3)	2,634 (26.1)
Ethnicity	White	84 (41.4)	9,092 (90.1)
	Non-White	27 (13.3)	1,003 (9.9)
	Missing data	92 (45.3)	
Marital status	Married/cohabiting	139 (68.5)	7,484 (74.1)
	Single/Divorced/Widowed	60 (29.6)	2,611 (25.9)
	Missing data	4 (2.0)	
**Health behaviours**			
Smoking	Never smoker	82 (40.4)	5,021 (49.7)
	Former smoker	48 (23.6)	3,246 (32.2)
	Current smoker	61 (30.0)	1,828 (18.1)
	Missing data	12 (5.9)	
Mean (SD) hours of MVPA per week		3.90 (5.0)	3.86 (4.3)
	Missing data	95 (46.8)	
Alcohol consumption	0 units/week	56 (27.6)	1,834 (18.2)
	1-14 units/week	97 (47.8)	5,858 (58.0)
	>14 units/week	34 (16.7)	2,403 (23.8)
	Missing data	16 (7.9)	
Fruit/vegetable consumption	< Once/day	89 (43.8)	4,212 (41.7)
	≥ Once/day	108 (53.2)	5,883 (58.3)
	Missing data	6 (3.0)	

Abbreviations: MVPA, Moderate and Vigorous Physical Activity; SD, Standard Deviation.
Values are N (% of excluded participants, % of included participants) unless stated otherwise.

Supplementary Table C. Association between multimorbidity (≥ 2 chronic conditions) and subsequent risk of dementia as a function of age at onset of multimorbidity, excluding Parkinson's disease from the conditions considered in the definition of multimorbidity[a]

Multimorbidity status	N Dementia/Total	Incidence rate per 1,000 person years	Incidence rate difference (95% CI) per 1,000 person years	Model 1 HR (95% CI)	Model 2 HR (95% CI)	Model 3 HR (95% CI)
**At age 55 years (Median (IQR) follow-up = 19.6 (15.6, 25.1) years)**						
0 or 1 chronic condition	587/9,282	3.10	0 (reference)	1 (reference)	1 (reference)	1 (reference)
Multimorbidity	51/655	4.67	1.56 (0.62 to 2.77)	2.51 (1.87 to 3.35)	2.47 (1.84 to 3.30)	2.44 (1.82 to 3.26)
**At age 60 years (Median (IQR) follow-up = 14.8 (10.8, 20.2) years)**						
0 or 1 chronic condition	542/8,498	4.04	0 (reference)	1 (reference)	1 (reference)	1 (reference)
Multimorbidity between 55-60	41/641	4.64	0.60 (-0.37 to 1.91)	1.45 (1.05 to 1.99)	1.40 (1.02 to 1.93)	1.39 (1.01 to 1.91)
Multimorbidity before age 55	50/621	6.46	2.42 (1.09 to 4.12)	2.57 (1.91 to 3.45)	2.52 (1.88 to 3.39)	2.46 (1.83 to 3.31)
p for trend				*<0.001*	*<0.001*	*<0.001*
**At age 65 years (Median (IQR) follow-up = 10.1 (6.1, 15.4) years)**						
0 or 1 chronic condition	468/7,448	5.60	0 (reference)	1 (reference)	1 (reference)	1 (reference)
Multimorbidity between 60-65	63/855	7.33	1.73 (0.53 to 3.25)	1.52 (1.17 to 1.98)	1.50 (1.15 to 1.95)	1.49 (1.14 to 1.94)
Multimorbidity between 55-60	39/601	6.84	1.24 (-0.24 to 3.22)	1.49 (1.08 to 2.07)	1.44 (1.03 to 2.00)	1.40 (1.01 to 1.95)
Multimorbidity before age 55	45/578	9.45	3.85 (1.79 to 6.51)	2.56 (1.88 to 3.49)	2.51 (1.84 to 3.43)	2.45 (1.79 to 3.35)
p for trend				*<0.001*	*<0.001*	*<0.001*
**At age 70 years (Median (IQR) follow-up = 6.7 (3.0, 11.4) years)**						
0 or 1 chronic condition	353/5,323	8.46	0 (reference)	1 (reference)	1 (reference)	1 (reference)
Multimorbidity between 65-70	70/890	10.65	2.19 (0.70 to 4.06)	1.31 (1.01 to 1.69)	1.30 (1.003 to 1.68)	1.27 (0.98 to 1.65)
Multimorbidity between 60-65	50/684	10.75	2.29 (0.38 to 4.78)	1.44 (1.07 to 1.94)	1.40 (1.04 to 1.89)	1.38 (1.02 to 1.86)
Multimorbidity between 55-60	32/469	10.97	2.51 (-0.10 to 6.10)	1.49 (1.04 to 2.14)	1.41 (0.98 to 2.04)	1.36 (0.94 to 1.96)
Multimorbidity before age 55	37/417	17.38	8.92 (4.63 to 14.56)	2.79 (1.98 to 3.93)	2.70 (1.92 to 3.81)	2.59 (1.84 to 3.66)
p for trend				*<0.001*	*<0.001*	*<0.001*
**Overall (Multimorbidity & covariates as time-varying measures; Median (IQR) follow-up = 31.7 (31.1, 32.6) years)**						
0 or 1 chronic condition	194/4,859	1.33	0 (reference)	1 (reference)	1 (reference)	1 (reference)
Multimorbidity	445/5,236	2.87	1.55 (1.47 to 1.63)	2.37 (1.99 to 2.82)	2.36 (1.98 to 2.82)	2.29 (1.92 to 2.73)

[a]Multimorbidity is defined as 2 or more chronic conditions out of the following 12 conditions: coronary heart disease, stroke, heart failure, diabetes, hypertension, cancer, chronic kidney disease, chronic obstructive pulmonary disease, liver disease, depression, mental disorders, and arthritis/ rheumatoid arthritis.

Model 1: stratified on birth-cohort (5-year groups) and adjusted for age (as time scale) and sex. Model 2: Model 1 + ethnicity, marital status, and education. Model 3: Model 2 + health behaviours (smoking, alcohol consumption, physical activity, and diet). Covariates measurement is concurrent to the measure of multimorbidity.

Supplementary Table D. Characteristics of participants as a function of mortality status at end of follow-up

Participant characteristics at baseline (1985-1998)		Mortality status at March 2019 Deceased	Alive
**N**		**N= 2,136**	**N= 7,959**
Sex	Men	1,396 (65.4)	5,401 (67.9)
	Women	740 (34.6)	2,558 (32.1)
Mean (SD) age, years		48.5 (5.7)	43.9 (5.8)
Education	Low secondary school or lower	1,179 (55.2)	3,596 (45.2)
	High school diploma	546 (25.6)	2,140 (26.9)
	University degree or higher	411 (19.2)	2,223 (27.9)
Ethnicity	White	1,911 (89.5)	7,181 (90.2)
	Non-White	225 (10.5)	778 (9.8)
Marital status	Married/cohabiting	1,524 (71.3)	5,960 (74.9)
	Single/Divorced/Widowed	612 (28.7)	1,999 (25.1)
**Health behaviours**			
Smoking	Never smoker	851 (39.8)	4,170 (52.4)
	Former smoker	662 (31.0)	2,584 (32.5)
	Current smoker	623 (29.2)	1,205 (15.1)
Mean (SD) hours of MVPA per week		3.7 (4.3)	3.9 (4.3)
Alcohol consumption	0 units/week	472 (22.1)	1,362 (17.1)
	1-14 units/week	1,158 (54.2)	4,700 (59.1)
	>14 units/week	506 (23.7)	1,897 (23.8)
Fruit/vegetable consumption	< Once/day	971 (45.5)	3,241 (40.7)
	≥ Once/day	1,165 (54.5)	4,718 (59.3)
**Chronic conditions at the end of follow-up**			
Coronary heart disease		734 (34.4)	1,531 (19.2)
Stroke		229 (10.7)	271 (3.4)
Heart failure		314 (14.7)	318 (4.0)
Diabetes		439 (20.6)	1,303 (16.4)
Hypertension		1,468 (68.7)	5,196 (65.3)
Cancer		1,036 (48.5)	1,220 (15.3)
Chronic kidney disease		227 (10.6)	362 (4.5)
Chronic obstructive pulmonary disease		242 (11.3)	312 (3.9)
Liver disease		155 (7.3)	146 (1.8)
Depression		339 (15.9)	979 (12.3)
Mental disorders		149 (7.0)	424 (5.3)
Parkinson's disease		86 (4.0)	98 (1.2)
Arthritis/Rheumatoid arthritis		294 (13.8)	1,336 (16.8)
**Multimorbidity status at the end of follow-up**			
0 or 1 chronic condition		570 (26.7)	4,155 (52.2)
Multmorbidity (≥ 2 chronic conditions)		1,566 (73.3)	3,804 (47.8)

Abbreviations: MVPA, Moderate and Vigorous Physical Activity; SD, Standard Deviation.
Values are numbers (percentages) unless stated otherwise.

Supplementary Table E. Association between chronic diseases considered individually and subsequent risk of mortality

	Prevalent at 55 years[a]	Prevalent at 60 years[a]	Prevalent at 65 years[a]	Prevalent at 70 years[a]	Overall[b]
	HR (95% CI)	HR (95% CI)	HR (95% CI)	HR (95% CI)	HR (95% CI)
Coronary heart disease	1.94 (1.64 to 2.30)	1.78 (1.54 to 2.06)	1.58 (1.37 to 1.81)	1.41 (1.22 to 1.63)	1.87 (1.68 to 2.08)
Stroke	2.01 (0.90 to 4.50)	2.37 (1.37 to 4.10)	2.10 (1.36 to 3.23)	2.41 (1.72 to 3.38)	4.41 (3.67 to 5.30)
Heart failure	NA	6.72 (3.47 to 13.02)	3.92 (2.35 to 6.55)	3.18 (2.27 to 4.44)	1.65 (1.46 to 1.87)
Diabetes	1.85 (1.46 to 2.33)	1.80 (1.49 to 2.17)	1.65 (1.39 to 1.95)	1.71 (1.44 to 2.02)	1.89 (1.68 to 2.12)
Hypertension	1.42 (1.29 to 1.56)	1.43 (1.31 to 1.58)	1.29 (1.17 to 1.43)	1.34 (1.19 to 1.51)	1.67 (1.52 to 1.85)
Cancer	2.70 (2.13 to 3.40)	2.25 (1.84 to 2.76)	2.28 (1.92 to 2.70)	2.01 (1.70 to 2.38)	9.03 (8.06 to 10.13)
Chronic kidney disease	NA	6.53 (2.70 to 15.80)	4.20 (1.73 to 10.19)	8.00 (5.04 to 12.70)	6.21 (5.15 to 7.48)
COPD	12.92 (5.30 to 31.52)	8.25 (4.62 to 14.74)	4.80 (3.05 to 7.55)	2.72 (1.88 to 3.92)	4.45 (3.75 to 5.27)
Liver disease	NA	11.11 (5.92 to 20.87)	3.88 (2.07 to 7.26)	2.76 (1.56 to 4.90)	8.38 (6.61 to 10.63)
Depression	1.10 (0.87 to 1.40)	1.11 (0.90 to 1.38)	1.03 (0.83 to 1.27)	1.17 (0.95 to 1.45)	1.79 (1.57 to 2.03)
Mental disorders	4.00 (1.78 to 8.99)	2.27 (1.07 to 4.81)	2.12 (1.17 to 3.86)	1.64 (0.92 to 2.90)	3.17 (2.64 to 3.82)
Parkinson’s disease	NA	NA	8.24 (3.68 to 18.49)	10.71 (5.50 to 20.86)	6.49 (5.04 to 8.35)
Arthritis/Rheumatoid arthritis	NA	1.23 (0.71 to 2.14)	1.35 (0.97 to 1.89)	1.36 (1.07 to 1.73)	1.52 (1.33 to 1.74)

Abbreviations: COPD, Chronic obstructive pulmonary disease; NA, Not Applicable as insufficient number of cases (<5) to allow analysis.

[a]Four separate analyses were run for prevalent disease at age 55, 60, 65, and 70 years with median follow-up of 19.9, 15.0, 10.3, and 6.9 years, respectively. All chronic diseases were examined in separate models.

[b]All chronic diseases were studied in separate models, and covariates were entered as time-varying measures. The median follow-up was 31.7 years.

Analyses were stratified on birth-cohort (5-year groups) and adjusted for age (as time scale), sex, ethnicity, education, marital status, and health behaviours (smoking, alcohol consumption, physical activity, and diet). Covariates measurement is concurrent to the measure of chronic diseases.

Supplementary Table F. Association between multimorbidity (≥ 2 chronic conditions) as a function of age at onset of multimorbidity and subsequent risk of mortality[a]

Multimorbidity status	N Mortality/Total	Incidence rate per 1,000 person years	Incidence rate difference (95% CI) per 1,000 person years	Model 1 HR (95% CI)	Model 2 HR (95% CI)	Model 3 HR (95% CI)
**At age 55 years (Median (IQR) follow-up= 19.9 (15.7, 25.5) years)**						
0 or 1 chronic condition	1,804/9,282	9.45	0 (reference)	1 (reference)	1 (reference)	1 (reference)
Multimorbidity	174/655	15.72	6.26 (4.45 to 8.33)	2.29 (1.96 to 2.68)	2.28 (1.94 to 2.67)	2.24 (1.91 to 2.63)
**At age 60 years (Median (IQR) follow-up= 15.0 (10.9, 20.5) years)**						
0 or 1 chronic condition	1,495/8,497	11.01	0 (reference)	1 (reference)	1 (reference)	1 (reference)
Multimorbidity between 55-60	167/642	18.66	7.65 (5.48 to 10.14)	1.99 (1.70 to 2.34)	2.03 (1.72 to 2.38)	1.99 (1.69 to 2.34)
Multimorbidity before age 55	142/621	18.07	7.06 (4.77 to 9.72)	2.29 (1.92 to 2.73)	2.29 (1.92 to 2.73)	2.27 (1.90 to 2.70)
p for trend				*<0.001*	*<0.001*	*<0.001*
**At age 65 years (Median (IQR) follow-up= 10.3 (6.2, 15.7) years)**						
0 or 1 chronic condition	1,134/7,444	13.34	0 (reference)	1 (reference)	1 (reference)	1 (reference)
Multimorbidity between 60-65	171/858	19.49	6.15 (4.10 to 8.50)	1.65 (1.41 to 1.94)	1.66 (1.41 to 1.95)	1.62 (1.38 to 1.91)
Multimorbidity between 55-60	127/602	21.86	8.52 (5.65 to 11.87)	1.92 (1.60 to 2.31)	1.96 (1.63 to 2.36)	1.92 (1.59 to 2.31)
Multimorbidity before age 55	100/578	20.57	7.23 (4.16 to 10.87)	2.15 (1.75 to 2.64)	2.17 (1.76 to 2.66)	2.13 (1.73 to 2.63)
p for trend				*<0.001*	*<0.001*	*<0.001*
**At age 70 years (Median (IQR) follow-up= 6.9 (3.1, 11.7) years)**						
0 or 1 chronic condition	710/5,319	16.62	0 (reference)	1 (reference)	1 (reference)	1 (reference)
Multimorbidity between 65-70	201/894	29.65	13.02 (10.27 to 16.15)	1.83 (1.56 to 2.14)	1.85 (1.58 to 2.16)	1.80 (1.54 to 2.11)
Multimorbidity between 60-65	110/683	23.04	6.42 (3.52 to 9.88)	1.55 (1.27 to 1.90)	1.55 (1.26 to 1.89)	1.57 (1.28 to 1.92)
Multimorbidity between 55-60	91/470	30.33	13.71 (9.00 to 19.35)	2.06 (1.65 to 2.56)	2.12 (1.70 to 2.64)	2.09 (1.67 to 2.60)
Multimorbidity before age 55	62/417	28.19	11.57 (6.19 to 18.25)	2.20 (1.70 to 2.86)	2.23 (1.72 to 2.91)	2.19 (1.68 to 2.85)
p for trend				*<0.001*	*<0.001*	*<0.001*
**Overall (Multimorbidity & covariates as time-varying; Median (IQR) follow-up= 31.7 (31.1, 32.7) years)**						
0 or 1 chronic condition	570/4,725	3.97	0 (reference)	1 (reference)	1 (reference)	1 (reference)
Multimorbidity	1,566/5,370	9.81	5.83 (5.67 to 5.99)	5.21 (4.68 to 5.80)	5.25 (4.72 to 5.84)	4.86 (4.36 to 5.41)
**Overall, without cancer (Multimorbidity & covariates as time-varying; Median (IQR) follow-up= 31.7 (31.1, 32.7) years)**						
0 or 1 chronic condition	874/5,417	5.39	0 (reference)	1 (reference)	1 (reference)	1 (reference)
Multimorbidity	1,262/4,678	8.96	3.58 (3.44 to 3.72)	3.57 (3.24 to 3.93)	3.59 (3.26 to 3.95)	3.13 (3.01 to 3.65)

[a] Multimorbidity defined as 2 or more chronic conditions out of the following 13 conditions: coronary heart disease, stroke, heart failure, diabetes, hypertension, cancer, chronic kidney disease, chronic obstructive pulmonary disease, liver disease, depression, mental disorders, Parkinson's disease, and arthritis/rheumatoid arthritis. Covariates measurement is concurrent to the measure of multimorbidity.
Model 1: stratified on birth-cohort (5-year groups).and adjusted for age (as time scale) and sex.
Model 2: Model 1 + ethnicity, marital status, and education.
Model 3: Model 2 + health behaviors (smoking, alcohol consumption, physical activity, and diet).

Supplementary Table G. Association between number of chronic conditions (≤1, 2, and ≥3) and subsequent risk of mortality[a]

Multimorbidity	N Mortality /Total	Incidence rate per 1,000 person years	Incidence rate difference (95% CI) per 1000 person years	Model 1 HR (95% CI)	Model 2 HR (95% CI)	Model 3 HR (95% CI)
**At age 55 years (Median (IQR) follow-up= 19.9 (15.7, 25.5) years)**						
0 or 1 chronic condition	1,804/9,282	9.45	0 (reference)	1 (reference)	1 (reference)	1 (reference)
2 chronic conditions	145/571	14.74	5.29 (3.42 to 7.45)	2.11 (1.78 to 2.51)	2.11 (1.78 to 2.51)	2.10 (1.77 to 2.49)
≥ 3 chronic conditions	29/84	23.45	14.00 (6.69 to 23.78)	4.03 (2.79 to 5.84)	3.84 (2.65 to 5.56)	3.43 (2.37 to 4.99)
**At age 60 years (Median (IQR) follow-up= 15.0 (10.9, 20.5) years)**						
0 or 1 chronic condition	1,495/8,497	11.01	0 (reference)	1 (reference)	1 (reference)	1 (reference)
2 chronic conditions	235/1,004	17.04	6.04 (4.48 to 7.79)	1.88 (1.64 to 2.16)	1.91 (1.66 to 2.20)	1.89 (1.65 to 2.18)
≥ 3 chronic conditions	74/259	24.52	13.52 (8.80 to 19.21)	3.58 (2.82 to 4.54)	3.55 (2.80 to 4.51)	3.41 (2.68 to 4.33)
**At age 65 years (Median (IQR) follow-up= 10.3 (6.2, 15.7) years)**						
0 or 1 chronic condition	1,134/7,444	13.34	0 (reference)	1 (reference)	1 (reference)	1 (reference)
2 chronic conditions	280/1,495	18.80	5.45 (4.08 to 6.99)	1.63 (1.43 to 1.86)	1.65 (1.44 to 1.88)	1.62 (1.42 to 1.85)
≥ 3 chronic conditions	118/543	25.94	12.60 (8.89 to 16.92)	2.69 (2.22 to 3.26)	2.72 (2.24 to 3.30)	2.63 (2.17 to 3.20)
**At age 70 years (Median (IQR) follow-up= 6.9 (3.1, 11.7) years)**						
0 or 1 chronic condition	710/5,319	16.62	0 (reference)	1 (reference)	1 (reference)	1 (reference)
2 chronic conditions	281/1,639	23.81	7.19 (5.69 to 8.88)	1.53 (1.33 to 1.75)	1.54 (1.34 to 1.77)	1.54 (1.34 to 1.77)
≥ 3 chronic conditions	183/825	36.95	20.33 (16.37 to 24.81)	2.67 (2.27 to 3.15)	2.71 (2.29 to 3.19)	2.60 (2.20 to 3.07)
**Overall (Multimorbidity & covariates as time-varying; Median (IQR) follow-up= 31.7 (31.1, 32.7) years)**						
0 or 1 chronic condition	570/4,725	3.97	0 (reference)	1 (reference)	1 (reference)	1 (reference)
2 chronic conditions	555/2,425	7.72	3.74 (3.43 to 4.07)	3.29 (2.88 to 3.75)	3.28 (2.87 to 3.75)	3.21 (2.81 to 3.67)
≥ 3 chronic conditions	1,011/2,945	11.52	7.55 (7.17 to 7.94)	8.67 (7.66 to 9.82)	8.83 (7.79 to 10.01)	8.12 (7.16 to 9.21)
**Overall, without cancer (Multimorbidity & covariates as time-varying; Median (IQR) follow-up= 31.7(31.1, 32.7) years)**						
0 or 1 chronic condition	874/5,417	5.39	0 (reference)	1 (reference)	1 (reference)	1 (reference)
2 chronic conditions	466/2,258	6.84	1.45 (1.20 to 1.73)	1.78 (1.57 to 2.03)	1.78 (1.56 to 2.02)	1.73 (1.52 to 1.98)
≥ 3 chronic conditions	796/2,420	10.95	5.57 (5.17 to 5.99)	5.63 (4.97 to 6.39)	5.64 (4.97 to 6.40)	5.09 (4.47 to 5.78)

[a]Chronic conditions considered were: coronary heart disease, stroke, heart failure, diabetes, hypertension, cancer, chronic kidney disease, chronic obstructive pulmonary disease, liver disease, depression, mental disorders, Parkinson's disease, and arthritis/rheumatoid arthritis. Model 1: stratified on birth-cohort (5-year groups) and adjusted for age (as time scale) and sex; Model 2: Model 1 + ethnicity, marital status, and education; Model 3: Model 2 + health behaviors (smoking, alcohol consumption, physical activity, and diet).

Supplementary Figure A. Flow-chart

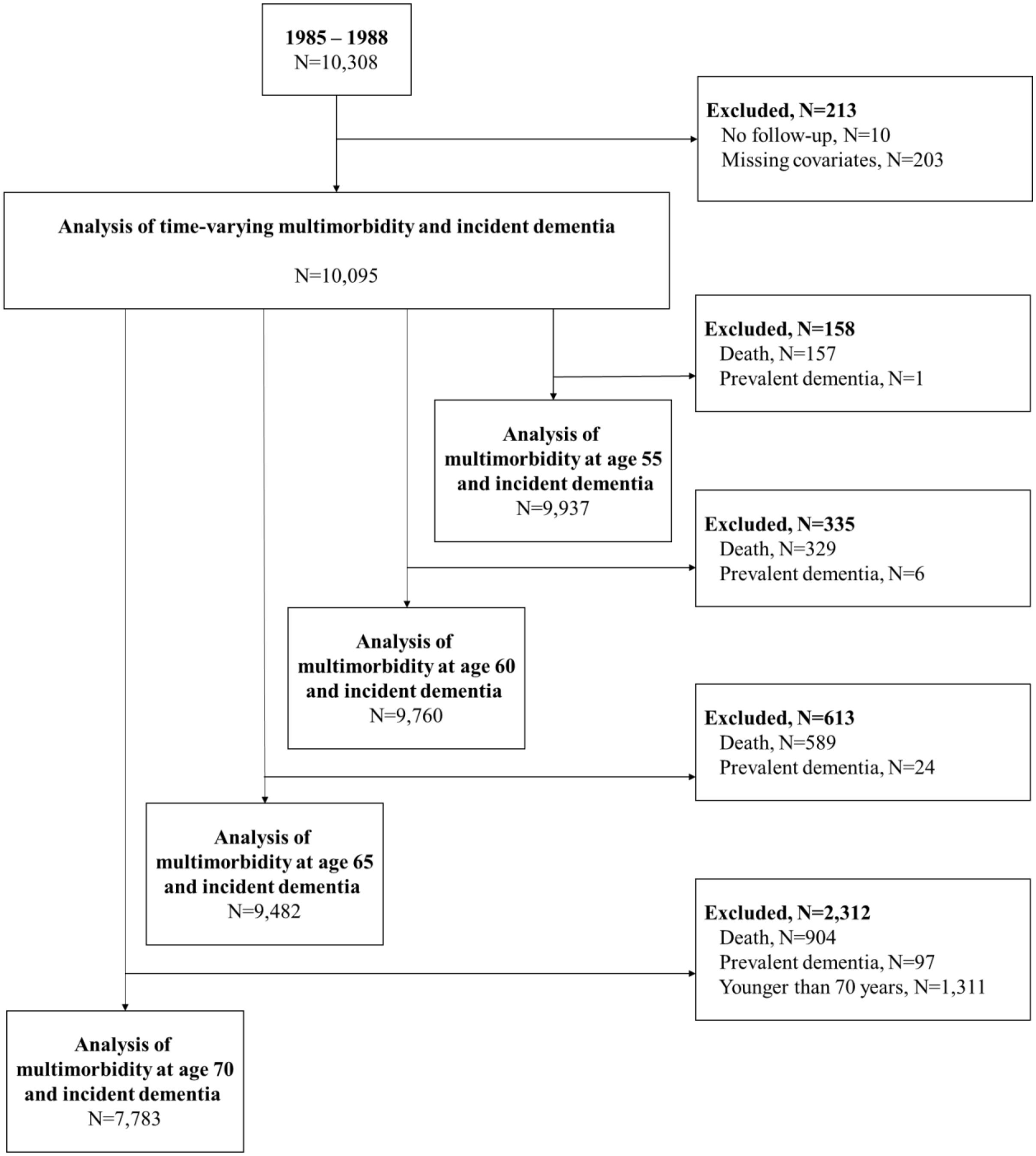

Supplementary Figure B. Prevalence of individual chronic diseases at age 55, 60, 65, and 70 years[a]

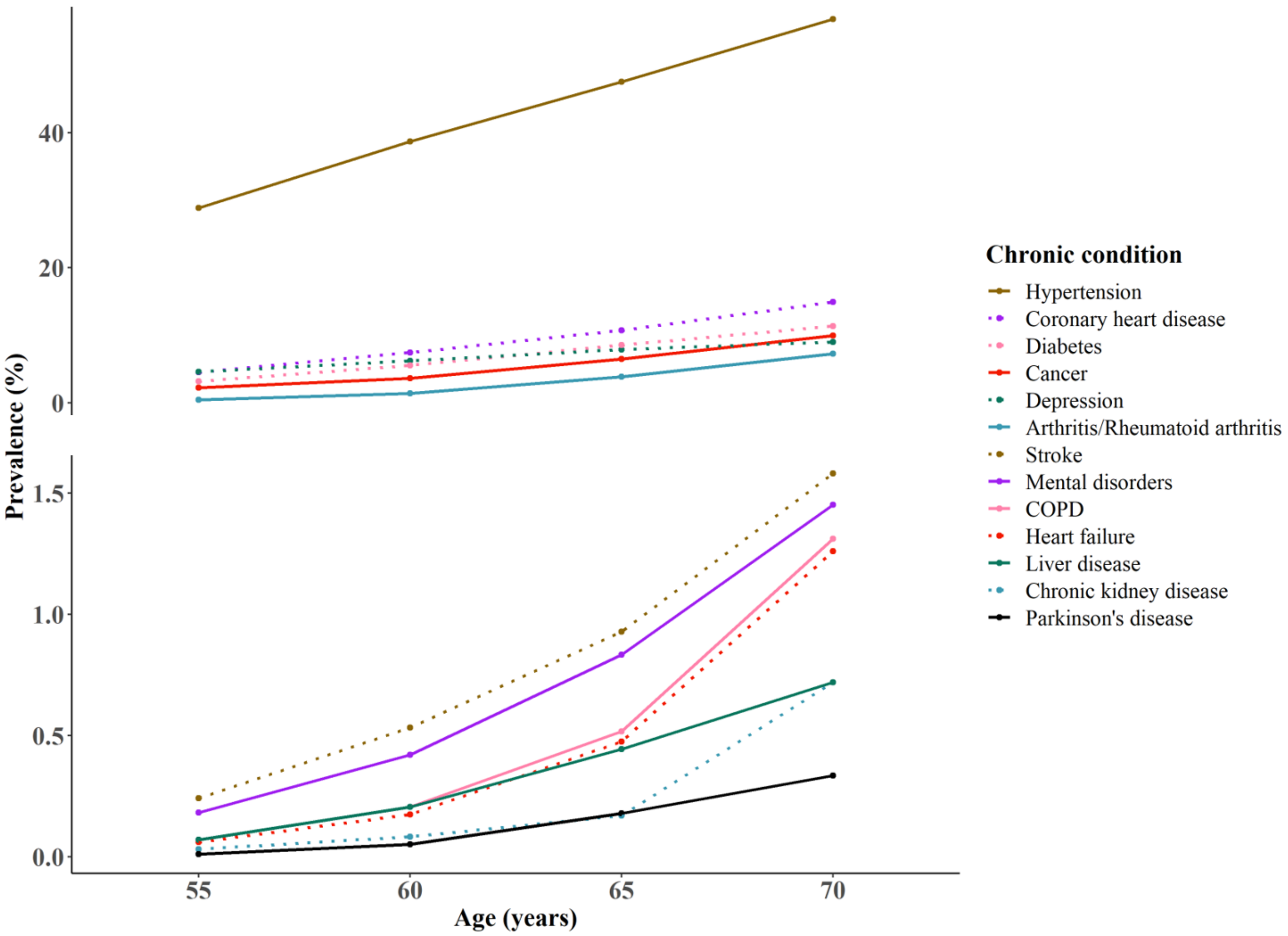

[a]The y-axis of the figure is split in two to distinguish between conditions that are more prevalent by placing them at the top (hypertension, coronary heart disease, diabetes, cancer, depression, and arthritis/rheumatoid arthritis, top frame) and others in the bottom panel (stroke, mental disorders, COPD, heart failure, liver disease, chronic kidney disease, Parkinson's disease).

Supplementary Figure C. Number of dementia cases and total number of participants with the corresponding dyads

	Coronary heart disease	Stroke	Heart failure	Diabetes	Hypertension	Cancer	Chronic kidney disease	COPD	Liver disease	Depression	Mental disorders	Parkinson's disease
Stroke	27/170											
Heart failure	40/376	13/64										
Diabetes	63/609	24/120	27/195									
Hypertension	158/1,877	56/365	56/547	137/1,441								
Cancer	21/478	8/98	9/143	25/398	82/1,503							
Chronic kidney disease	34/253	12/54	21/175	29/217	60/502	11/181						
COPD	26/214	5/41	22/124	13/140	52/418	13/162	12/80					
Liver disease	5/91	2/19	5/41	4/80	15/217	2/114	1/40	6/42				
Depression	50/326	22/67	17/91	41/293	126/914	34/284	18/87	20/126	2/50			
Mental disorders	27/140	9/36	14/52	21/127	73/368	19/133	13/48	10/57	2/28	50/286		
Parkinson's disease	14/38	1/12	3/14	10/31	29/105	7/25	3/10	1/4	0/2	11/33	7/22	
Arthritis/ Rheumatoid arthritis	43/486	18/90	20/188	31/336	112/1,253	24/395	18/187	20/160	4/58	47/272	33/150	8/32

Abbreviations: COPD, Chronic obstructive pulmonary disease.
